# Impact of the workforce allocation on the technical performance of mental health services: the collective case of Helsinki-Uusimaa (Finland)

**DOI:** 10.1186/s12961-023-01061-y

**Published:** 2023-10-23

**Authors:** Diego Diaz-Milanes, Nerea Almeda, Mencia R. Gutierrez-Colosia, Carlos R. Garcia-Alonso, Minna Sadeniemi, Luis Salvador-Carulla

**Affiliations:** 1 Department of Quantitative Methods, Universidad Loyola Andalucía, Avenida de las Universidades, S/N, Dos Hermanas, Seville, 41704 Cordova, Spain; 2 Institute of Health Research, University of Canberra, Canberra, Australia; 3 Department of Psychology, Universidad Loyola Andalucía, Seville, Spain; 4 Varma Mutual Pension Insurance Company, Varma, Finland; 5 Health Information Systems Group (SICA-CTS-553), University of Cadiz, Cadiz, Spain

**Keywords:** Mental healthcare, Finland, Relative technical efficiency, Decision support system, Services management, Workforce

## Abstract

**Background:**

Long-term mental health (MH) policies in Finland aimed at investing in community care and promoting reforms have led to a reduction in the number of psychiatric hospital beds. However, most resources are still allocated to hospital and community residential services due to various social, economic and political factors. Despite previous research focussing on the number and cost of these services, no study has evaluated the emerging patterns of use, their technical performance and the relationship with the workforce structure.

**Objective:**

The purpose of this study was to observe the patterns of use and their technical performance (efficiency) of the main types of care of MH services in the Helsinki-Uusimaa region (Finland), and to analyse the potential relationship between technical performance and the corresponding workforce structure.

**Methods:**

The sample included acute hospital residential care, non-hospital residential care and outpatient care services. The analysis was conducted using regression analysis, Monte Carlo simulation, fuzzy inference and data envelopment analysis.

**Results:**

The analysis showed a statistically significant linear relationship between the number of service users and the length of stay, number of beds in non-hospital residential care and number of contacts in outpatient care services. The three service types displayed a similar pattern of technical performance, with high relative technical efficiency on average and a low probability of being efficient. The most efficient acute hospital and outpatient care services integrated multidisciplinary teams, while psychiatrists and nurses characterized non-hospital residential care.

**Conclusions:**

The results indicated that the number of resources and utilization variables were linearly related to the number of users and that the relative technical efficiency of the services was similar across all types. This suggests homogenous MH management with small variations based on workforce allocation. Therefore, the distribution of workforce capacity should be considered in the development of effective policies and interventions in the southern Finnish MH system.

**Supplementary Information:**

The online version contains supplementary material available at 10.1186/s12961-023-01061-y.

## Background

To meet population mental health (MH) needs, planners aim to design the most efficient and effective facility structures and workforce teams to provide quality care in both hospital- and community-based services. The balanced care model [1] describes the basic elements, including workforce structure, for organizing a feasible MH care depending on the income levels of the countries, regions, etc. This model offers a framework to optimize MH care provision, prioritizing community care while preserving the minimum hospital care required (depending on the specific characteristics of the real MH ecosystem). As a paradigm, the balanced care model can be used by researchers and policymakers to develop policies and strategies for shifting resources from hospital-based services to community-based services, which provide rehabilitative practices and social facilities and promote the acceptance and inclusion of users by their families and communities. Furthermore, it also aims to improve the technical performance of community-based staff by emphasizing multidisciplinary teamwork, striking a balance between patient control and independence, promoting evidence-based treatments, recognizing the importance of families and their therapeutic potential, and finding equilibrium in resource allocation and service provision, among other goals [2, 3].

The evolution of MH care systems worldwide and the application of this model have been strongly influenced by contextual factors. This resulted in a wide range of different types of MH settings across the world, meaning that no single model can be universally applied [4]. Nonetheless, this paradigm can be useful as a framework for analysing the performance of specific MH systems, particularly in terms of achieving the optimal balance of care [5] and underscoring the importance of an effective workforce management for improving MH care technical performance, especially concerning family or social support availability [6].

The technical performance of healthcare systems typically involves the analysis of service availability metrics (e.g. rates of hospital beds, staff in acute wards, etc.), resource utilization (e.g. length of stay, re-hospitalization rates, etc.) and quality-of-care assessment metrics [7]. However, even an apparently robust indicator such as readmission rates has been questioned for not being comparable across hospitals and countries due to inconsistent measurement strategies and local system variability [8]. Furthermore, the use of the directories of services identified by their names could be a major source of bias in, for example, MH service research, and the disambiguation of the activity performed by these services requires a reassessment of their classification using international standard instruments [9].

The European Union funded REsearch on FINancing systems’ Effect on the quality of MENTal health care project (REFINEMENT; http://www.refinementproject.eu) compared differences in financing mechanisms, service provision and pathways of care in eight European countries to understand their impact on the quality and efficiency of regional MH systems. This project developed a decision support tool on the basis of the mapping of service provision in local areas (REMAST: REFINEMENT Mapping Services Tool). It combined a series of instruments such as the Description and Evaluation of Services and DirectoriEs for Long-Term Care (DESDE-LTC) for the standard classification of health services [10] and geographical information systems (GIS).

According to the DESDE-LTC codification system, to classify a MH service, it is necessary to fill out a specific questionnaire in a process in which external experts in MH management (including clinicians) are involved. They visit the services to analyse their structures and processes, drawing information from their databases and interviews, all to ensure comparability: two different MH services have the same code if they provide the same type of care to similar user’s sets.

Therefore, DESDE-LTC and REMAST were developed to facilitate the monitoring, reviewing and improving of regional MH systems, to compare the availability of resources and the care capacity across regions, to assess the accessibility to MH services, and to eventually facilitate efficiency analysis (as a technical performance indicator) [10]. This effort led to the production of healthcare databases with disambiguated information on regional service provision and available local resource utilization in several European health regions, such as Girona (Spain), Verona (Italy) and Helsinki-Uusimaa (Finland). This study was followed by another European study on hospitalization and readmission in six European countries [11].

The Helsinki and Uusimaa region had the second highest bed rates in health services and in social care out of the eight European health regions described in the REFINEMENT study [12, 13]. Residential services also represent a major area of health and social care provision. Most of the beds were in nursing homes with 24-h staff providing permanent care for people with severe mental disorders. The remainder were mainly beds in nursing homes with less intensive daily support.

The long-lasting policy of investing in community care and the reform process in Finland [14, 15] reduced the number of psychiatric hospital beds by more than 75% between the 1970s and 2010s [16]. However, there were still 48 hospital beds/100,000 inhabitants in Finland in 2021 [17], mainly in housing services owned and managed by private companies or third-sector providers that offered their services to public MH areas such as municipalities [18–20]. The number of psychiatric inpatients remained stable during the 2000s (6 per 1000 inhabitants annually) [16], and the supply of intermediate psychiatric services did not increase during this period mainly due to a series of economic recessions [20]. Consequently, most of the resources continued to be allocated to hospital and community (non-hospital) residential services. Apart from financing constraints, the comparatively high allocation of resources to residential care in Helsinki-Uusimaa could be related to its high rates of one-person households [21], the weak role of the family in MH care in comparison with other countries in Europe [13, 18], and the division of the management of MH services across municipalities [19, 22], among other factors.

Previous studies have indicated that the municipal-level fragmentation of the Finnish MH system could result in uncontrolled growth of non-hospital residential and primary care provisions [19, 23]. The number of primary care psychiatric nurses is associated with less use of specialized outpatient care services but not with the use of hospital inpatient services [24]. This uneven pattern of care has been related to a lack of coordination among sectors (especially health and social sectors) and to link resource allocation by local governments to population values rather than to real population MH needs due to complex political, managerial and historical reasons [22, 23, 25].

Current reforms in Finland are oriented towards the integration of different care sectors to improve the continuity of comprehensive care, that is, through primary, specialized and social services, alongside the creation of autonomous bodies for the organization of social and health services in their corresponding geographical areas [26, 27]. Local and national planning should be based on data; the release of the REFINEMENT analyses between 2013 and 2015 coincided with the closure of three of the eight MH hospitals in this region. Most of these hospital users were relocated to nursing homes staffed 24 h a day [12]. Notably, the majority of nursing homes in Finland are private for-profit companies under public contracts and are highly profitable [28], and most of the resources are still allocated to residential care facilities, as their personnel costs are a significant cost driver in the southern Finnish MH system.

Previous studies have mainly focussed on the classification and description of MH services and workforce allocation but not on the impact of these factors on their actual performance. Therefore, this study aimed to study the patterns of use and their technical performance (efficiency) of the main types of care of MH services at the Helsinki-Uusimaa region (Finland), and to analyse the potential relationship between technical performance and their corresponding workforce structure.

## Methods

This is a collective case study of the typology and characteristics of care for MH in the Helsinki-Usimaa region using a mixed qualitative and quantitative approach. Collective case studies in healthcare analyse multiple individual cases or instances that share common characteristics or themes (in this case, care organizations, beds and professionals providing MH care in the region). These entities are examined collectively to gain a comprehensive understanding of the broader issue at hand, exploring similarities, differences and patterns of care provision to gain insights that can inform healthcare policies and quality improvement [29]. This type of study design is particularly useful when analysing complex systems within specific contextual conditions (ecosystem approach) [30]. It allows for the integration of sets of modelling tools to identify patterns and effectively summarize intricate information related to service provision [31].

### The REFINEMENT database of MH provisions in Helsinki-Uusimaa

The general characteristics, social and demographic, of the Helsinki-Uusimaa region, and the comparison of its MH service delivery system with those of other health regions in Europe, have been described in previous papers from the REFINEMENT Group [12, 13, 18]. It is a medium-sized and relatively homogeneous area from a MH management point of view.

The original REFINEMENT database is composed of structural information on services that provide health and social care for people experiencing MH problems in the area of Helsinki-Uusimaa and data on the related resource utilization. All services are classified according to their main types of care using DESDE-LTC [32] (translated in the Finnish version as “European Service Mapping Schedule-Research—ESMS-R [33]). This code (based on the expert evaluation of service activity) prevents ambiguity and facilitates modelling studies in health economics and comparative effectiveness for evidence-informed planning [9]. The full dataset was extracted from Sadeniemi et al. [18] and revised in 2018.

**Fig. 1:**
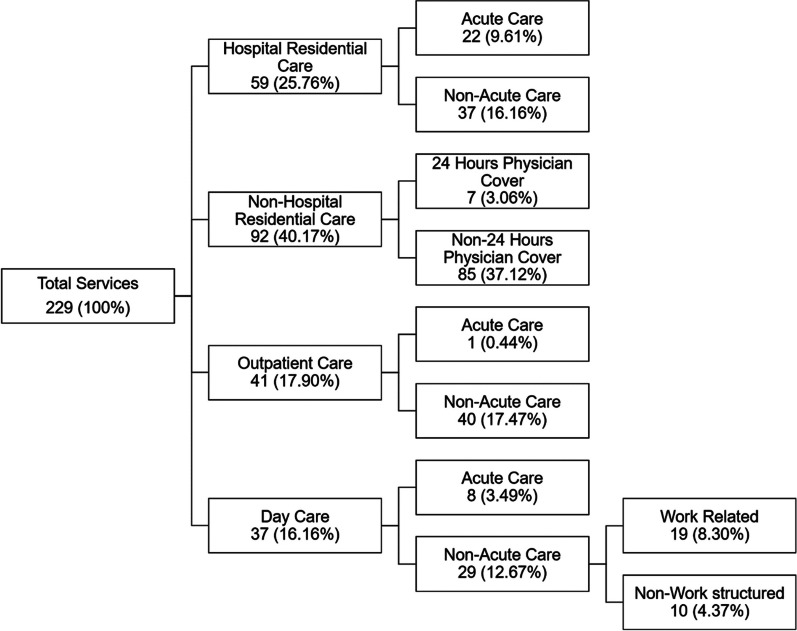
Distribution of service typologies according to DESDE-LTC

The original dataset included 229 MH services. Most of them provided non-hospital residential care (40.17%), followed by hospital residential care (25.76%), outpatient care (17.90%) and day care (16.16%) services, with non-acute care being predominant (Fig. 1). Most of the non-acute day care services were work related (8.30%), while most of the non-hospital residences were not covered by a physician 24 h a day (37.12%).

### Selected indicators for this study

Three MH service typologies were analysed in this study: T1: acute hospital residential care (DESDE-LTC codes R1 and R2), T2: non-hospital non-acute residential care (codes R9, R11, R12 and R13) and T3: non-acute outpatient care (codes O8, O9 and O10) (Table 1).

**Table 1:** Service types included in the analysis of technical performance according to DESDE-LTC [32]

Main type of care	DESDE-LTC code	Type of facilities
Residential care	Acute, hospital, 24 h physician cover, high intensity care (R1)	General hospitals, psychiatric hospitals and other specialized hospitals
	Acute, hospital, 24-h physician cover, medium intensity care (R2)	
	Non-acute, non-hospital, non-24-h physician cover, time limited, daily support (R9)	Residences, houses and therapeutic communities with various levels of support
	Non-acute, non-hospital, non-24-h physician cover, indefinite stay, 24-h support (R11)	
	Non-acute, non-hospital, non-24-h physician cover, indefinite stay, daily support (R12)	
	Non-acute, non-hospital, non-24-h physician cover, indefinite stay, lower support (R13)	
Outpatient care	Non-acute, non-mobile, high intensity (O8)	Community mental health teams, outpatient psychiatric clinics and single-handed psychiatrists and psychologists
	Non-acute, non-mobile, medium intensity (O9)	
	Non-acute, non-mobile, low intensity (O10)	

The selected indicators (26 from the REFINEMENT database) were classified as “inputs” (resources needed to provide MH care) or “outputs” (results or outcomes produced by the inputs) by the clinical experts participating in the REFINEMENT study in Finland and in Spain. Further information is provided in the REFINEMENT glossary of terms [34, 35] (Table 2). For hospital and non-hospital residential services, the input variable values were transformed into rates per *number of beds* (except for the *length of stay*, which is assessed in *days in the service* per *number of service users*). For outpatient care services, the rates were calculated per *number of service users*. For all the cases, the *number of service users*, *days of stay* and *contacts* were determined over a natural year. The reason for analysing rates per *number of users* or *beds* is to eliminate the service size effect into the performance analysis. Size is one of the most relevant sources of variability in this kind of studies because it usually is related to the number of users that look for a specific type of care in a geographical area. In many residential services the number of beds is usually used as a proxy for the number of users.

**Table 2:** Inputs and outputs selected to assess technical performance

Typologies	Inputs: workforce capacity	Outputs
Hospital acute (R1, R2)	Number of *psychiatrists*, *psychiatrists in training*, *nurses*, *psychologists*, *social workers* and *occupational therapists*, and finally, *other staff*. All rates per *number of beds*	• *Length of stay*: number of days in the service (rates per *number of service users*) • *Users*: number of service users (rates per *number of beds*) • *Contacts*: number of admissions (rates per *number of beds*)
Residential and non-hospital non-acute (R9, R11, R12, R13)	Number of* psychiatrists*, *nurses*, *social workers* and *occupational therapists*, and *other staff*. All rates per *number of beds*	• *Length of stay*: number of days in the service (rates per *number of service users*) • *Users*: number of service users (rates per *number of beds*)
Outpatient and non-acute (O8, O9, O10)	Number of *psychiatrists*, *psychiatrists in training*, *nurses*, *psychologists*, *social workers* and *occupational therapists*, and *other staff*. All rates per *number of service users*	• *Contacts*: number of contacts (rates per *number of service users*)

The data included in the analysis are available in Additional files 1, 2, 3.

### Data processing and analysis

#### Regression analysis

Regression analysis was firstly performed to identify potential patterns of use in the selected types of care. In this section of the study, strong relationships between the selected variables to assess MH service technical performance, if they exist, could highlight potential managerial guidelines and/or management profiles according to the social and economic structure of the municipalities and/or specific user characteristics. These specific patterns of use are very relevant to assuring the comparability of the sample. Taking into account that DESDE-LTC codes (types of care) are based on the service activities, sometimes specific managerial nuances can be hidden [36]. The resulting information of the regression analysis is then used in technical performance assessment by taking into account potential subsets and formalizing knowledge to carry out the “interpretation of variable values” process.

A linear regression model (using raw variables, no rates) was designed to estimate the placement capacity (*number of beds*) and the *length of stay* (days) in typologies T1 (hospital acute; R1 and R2) and T2 (residential and non-hospital non-acute; R9, R11, R12 and R13). In T3 (outpatient and non-acute; O8, O9 and O10), the *number of contacts* was also modelled using the same technique. The independent variable for all the regression models was the *number of service users*.

#### Knowledge discovery from data (KDD)

**Fig. 2:**
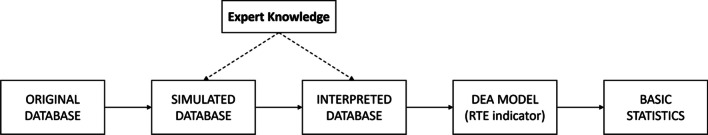
Diagram of the methods used to process and analyse the data

Knowledge discovery from databases combines data mining methods with different tools for extracting knowledge from data in, for example, performance analysis [37]. In KDD, methods from statistics, operational research, computational science, information systems, artificial intelligence (AI), visualization and association algorithms are used in a cooperative way to generate information from databases [38, 39]. In this study, an adaptation of the Efficient Decision Support-Mental Health (EDeS-MH) Decision Support System (DSS) was used [40] to assess the technical performance of MH care provision in the region of Helsinki-Uusimaa. The methodology used follows a healthcare ecosystem approach [41] and blended modelling techniques [39, 42], and it integrates operational techniques such as Monte Carlo simulation to incorporate randomness and uncertainty in the analysis, fuzzy inference for interpreting data (rates) according to the balanced care model, and data envelopment analysis (DEA) for technical performance assessment (relative technical efficiency, RTE) [40] (Fig. 2).

#### Monte Carlo simulation engine

To include the real structural and environmental randomness and uncertainty of the selected MH care system in the analysis, original data (Table 2) were transformed into standard statistical distributions [40]. Each statistical distribution was managed by a Monte Carlo simulation engine [43] and was selected by the group of experts to approximate the real system behaviour [44] according to the Expert-based Cooperative Analysis (EbCA) [42] methodology. Finally, triangular distributions were selected as their structures: the original value was considered the modal value, and the left/right values (range of the distribution) were calculated by decreasing/increasing the modal value by a percentage defined by experts (EbCA). For example, if the original variable value was 0.553, it was transformed into the following triangular distribution *T*[0.377, 0.553, 0.714]; in this case, the left/right values were calculated by decreasing/increasing the modal (original) value by 30%. The Monte Carlo simulation engine generated 1000 runs for each service (all considered decision-making units, DMUs, for the DEA). Nakayama’s error [45] was calculated for all the variables (inputs and outputs) as well as for RTE and technical performance scores. The maximum limit for the error was 2.5% on the corresponding RTE average [46]. This procedure has been extensively developed and explained in previous studies [40, 46].

#### Fuzzy inference engine

Variable values from the simulation engine were then interpreted according to expert knowledge formalized by EbCA methodology [42], taking the balanced care model [1, 2, 47] as the paradigm. All the simulated values were assessed in terms of appropriateness in accordance with this model, considering the typology and the profile of each MH service. This process integrates the local expert knowledge about when a variable value can be considered “appropriate”, according to the MH environment and the availability of resources, to provide quality of care.

The final variable value transformation was carried out by a fuzzy inference engine based on standard *IF … and … THEN …* rules (knowledge based). These rules activate the appropriate mathematical functions (linear monotone increasing/decreasing) for the mathematical transformation of variable values. For example, considering the variable *NurseR9* (non-standard input; *number of nurses* per 100,000 adults aged 18 years or older and inhabitants of an R9 service) can be considered “appropriate” within the range [0.1618, 0.1708]. Within this range, the higher the value is, the more appropriate it is. Outside it, original variable values are penalized (they are not appropriate according to the selected paradigm). If the value is on the left side of the range, the lower the original value is and the greater the penalization. If it is on the right side, the greater the original variable value is and the greater the penalization. In this specific example, if the original variable value is within the range, it is transformed according to Equ. (1):

1 $${x}_{\mathrm{transformed}}=\left({x}_{\mathrm{right}}+{x}_{\mathrm{left}}\right)-{x}_{\mathrm{original}}$$

where ${x}_{\mathrm{left}}=0.1618$, ${x}_{\mathrm{right}}=0.1708$ and ${x}_{\mathrm{original}}$ is the original variable value. The transformed values were used to design the final DEA model to be solved. This process has been explained in previous studies [40, 43, 46].

#### Relative technical efficiency (RTE) assessment

DEA is a robust non-parametric method based on linear programming, introduced by Charnes, Cooper and Rhodes in 1978 to evaluate the technical performance of a group of comparable DMUs. Over the years, DEA has been extensively used in the healthcare research domain, including mental health [35].

RTE can be defined as “*a service characteristic, which minimizes the inputs needed to achieve a given level of outcomes* – input orientation –*, or which maximizes the outcomes for a given level of inputs* – output orientation –*”* [4]. RTE assesses the balance between input (usually resources) consumption and output (outcomes directly related to the inputs) production in a set of comparable DMUs, for example, MH services [48]. The main concern of MH managers, to reduce input consumption without an output decrease, is the reason for selecting input orientation [46]. There is no evidence of a constant relationship between input consumption and output production, so the variable returns to scale DEA model was selected [44]. No weights were selected for the variables.

The RTE is “relative” because it compares the technical performance of each DMU to the others. The DMU that shows the best input/output balance has an RTE equal to 1 (maximum efficiency), while the rest of the DMUs have an RTE score of [0,1) (inefficient). An RTE equal to 0 means that the DMU is totally inefficient. When RTE is equal to 1 but there exists an input/output slack (they can be improved or optimized), the DMU is considered weakly efficient.

The integration of Monte Carlo simulation and fuzzy inference in DEA addresses the shortcomings of black box (in absence of expert knowledge), deterministic (without uncertainty and randomness) and linear analyses (severe limitations in the number of variables). In analysing real systems, simulation is needed to face uncertainty and randomness and expert knowledge is needed to interpret variable values (non-linear and discontinuous), and by using scenarios (expert-based variable combinations) the number of variables is no longer a problem.

#### Descriptive statistics for RTE scores

For all the selected services, the RTE probabilistic distributions were analysed (each Monte Carlo simulation run generates a different RTE score), including the following statistics: RTE average ($$\overline{\mathrm{RTE} }$$), RTE standard deviation ($$\overline{{\sigma }_{\mathrm{RTE}}}$$), RTE error ($$\overline{{\varepsilon }_{\mathrm{RTE}}}$$), RTE error percentage ($$\%\overline{{\varepsilon }_{\mathrm{RTE}}}$$), probability of being efficient (*P*_RTE==1_$$)$$), weakly efficient ($${P}_{\mathrm{RTE}\cong 1})$$) and inefficient ($${P}_{\mathrm{RTE}<1})$$), and finally, probability of RTE greater than 0.75 ($${P}_{\mathrm{RTE}>0.75})$$). Once the simulation process ends (1000 runs), the resulting RTE scores for statistical distribution (1000 values summarizing the selected randomness and uncertainty – Monte Carlo simulation engine – as well as expert knowledge – fuzzy inference engine) can be easily determined, and it is the primary source for calculating the RTE statistics. For an extensive explanation regarding these statistical indicators, see García-Alonso et al. [40].

### Statistical comparison for the workforce regarding RTE scores

RTE scores were classified into their respective quartiles. The DMUs in the first quartile had the lowest RTE scores, and those in the fourth quartile were the services with the highest RTE scores. The median workforce (gross and rates) of both groups (first and fourth quartiles) was compared by using the Mann–Whitney *U* test.

## Results

### Regression analysis and patterns of use

In typology T1 (acute hospital residential care; R1 and R2), the average *number of service users* per *number of beds* was 16.26 (SD = 9.46), but no linear relationship was found between the variables [*F*(1, 19) = 0.823; *p* = 0.376; *R*^2^ = 0.041]; likewise, no linear relationship was found between the *length of stay* and the *number of service users* [*F*(1, 19) = 1.119; *p* = 0.303; *R*^2^ = 0.233], with an average number of days per service user of 29.24 (SD = 29.03).

**Fig. 3:**
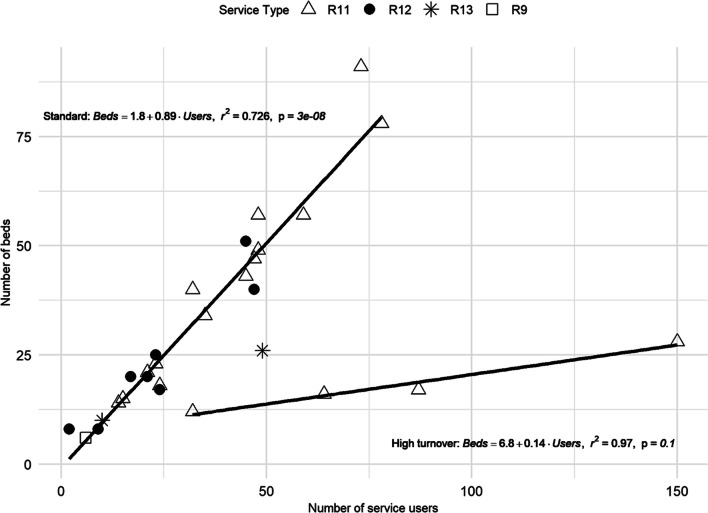
Linear regression comparing the *number of beds* and *number of service users* in the T2 typology. Service codes: R9, R11, R12 and R13. Patterns of use are highlighted

In typology T2 (non-acute non-hospital residential care; R9, R11, R12 and R13), two different patterns of use were found regarding the *number of beds* and *number of service users*. First, the standard pattern showed a significant linear relationship for most of its services, with an estimated coefficient equal to 1 (100 users, 100 beds), as expected considering the characteristics of T2 [*F*(1, 23) = 201; *p* < 0.001; *R*^2^ = 0.897] (Fig. 3).

The second pattern of use, high user turnover, was representative of some specific R11 services (Fig. 3), with an estimated coefficient of 0.14 (100 users, 14 beds) [*F*(1, 2) = 65.05; *p* = 0.015; *R*^2^ = 0.970]. There were two different R11 services in the analysed region.

**Fig. 4:**
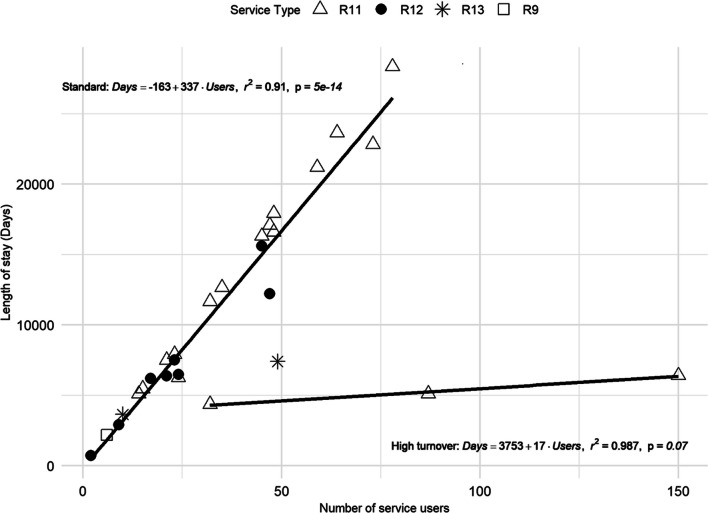
Linear regression of the *number of service users* and *length of stay* in non-hospital services. Service codes: R9, R11, R12 and R13. Patterns of use are highlighted

When analysing *length of stay* as a function of the *number of service users* at T2, two different patterns of use were again found. For the first one, a significant relationship between the selected variables was found [SD = 50.63; *F*(1, 24) = 242.5; *p* < 0.001; *R*^2^ = 0.91] (Fig. 4). In this pattern, the average *length of stay* was 335.2 days per user.

The second pattern of use showed an informative but non-significant model [SD = 50.33; *F*(1, 1) = 75.43; *p *= 0.073; *R*^2^ = 0.987]. Here, the average *length of stay* was 79.51 days per user. These services (mostly R11) are relatively scarce and showed a higher turnover of users during the year (Fig. 4).

**Fig. 5:**
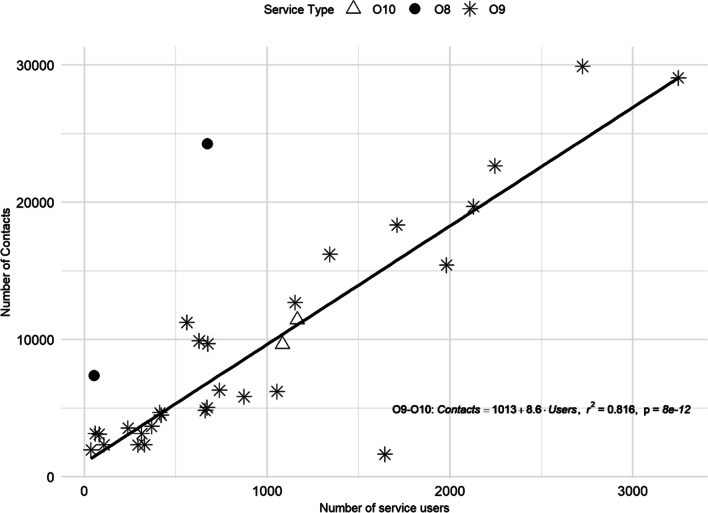
Linear regression of the *number of service users* and *number of contacts* in outpatient services. Service codes: O8, O9 and O10

Finally, in typology T3 (non-acute outpatient care; O8, O9 and O10), outpatient care, non-acute, non-mobile and high intensity services (O8) showed higher *number of contacts*/*number of service users* rates (*M* = 84.81; SD = 69.08) than lower intensity services (O9–O10) (*M* = 14.1; SD = 12.27). For outpatient care, non-acute, non-mobile and medium intensity (O9) and low intensity (O10) services, a significant linear relationship [*F*(1, 28) = 124.3; *p* < 0.001; *R*^2^ = 0.816] was found for these variables (Fig. 5).

### MH service technical performance

All the selected typologies showed similar basic statistics when RTE scores were analysed: very low or low probability of being efficient, very high or high average RTE, and finally, very high or high probability of RTE greater than 0.75 (Table 3). Differences appeared when specific types of care were analysed. In T1, the probability of being efficient was almost three times higher in R1 than in R2. In both types of care, the probability of having an RTE greater than 0.75 was very high.

**Table 3:** Relative technical efficiency (RTE) statistics per service type

Service type (DESDE codes)*	Efficiency average	Efficiency standard deviation	Efficiency error	Efficiency error percentage (%)	Probability of being efficient	Probability of being weakly efficient	Probability of being inefficient	Probability of RTE greater than 0.75
R1–R2	0.967	0.001	0.0008	0.0821	0.0622	0.013	0.9248	0.9992
R1	0.972	0.004	0.0026	0.2716	**0.1445**	0.0245	0.831	**0.992**
R2	0.966	0.001	0.0009	0.0885	**0.0531**	0.0117	0.9352	**1**
R9–R13	0.901	0.001	0.0009	0.0994	0.0074	0.0386	0.954	0.9121
R9	0.956	0.009	0.0068	0.7205	0.033	0.01	**0.957**	0.974
R11	**0.889**	0.002	0.0014	0.1601	0.0046	0.0034	**0.9921**	0.9121
R12	0.906	0.002	0.0013	0.1441	0.0105	0.005	**0.9845**	0.8941
R13	0.967	0.006	0.0042	0.4349	**0.008**	**0.5035**	**0.4885**	0.953
O8–O10	0.929	0.0009	0.0006	0.0667	0.0145	0.0572	0.9284	0.9488
O8	0.992	0.0006	0.0005	0.0457	**0.0073**	**0.6693**	**0.3233**	1
O9	0.921	0.0011	0.0008	0.087	0.0128	0.0045	**0.9827**	0.9395
O10	0.959	0.0027	0.0019	0.1983	0.0397	0.024	**0.9363**	1

*The definition of the DESDE codes is provided in Table 1. Elements underlined group the services of the same typology. Bold numbers highlight relevant differences

In T2, R13 services were considered very efficient (when considering weakly efficient simulations). In this typology, RTE on average (high in all the types of care included) could not be considered informative enough because it masked the differences in the probability of being inefficient.

In T3, O8 services showed a very high probability of being efficient (when weakly efficient solutions were included), as occurred in R13 (T2). Here and again, RTE on average (high) was not informative enough because it masked relevant differences in the probability of being inefficient.

### Relationship between workforce capacity and service technical performance

Service distribution in the RTE quartiles did not show any bias on the basis of the analysed types of care (Table 4).

**Table 4:** Number of services in each quartile on the basis of RTE scores

DESDE-LTC code	1st Quartile	2nd Quartile	3rd Quartile	4th Quartile
R1	1	0	0	1
R2	4	5	5	4
R9	0	0	1	0
R11	6	5	3	4
R12	2	2	2	2
R13	0	0	1	1
O8	0	0	1	2
O9	10	8	8	7
O10	0	2	0	1

Significant differences in the distribution of professionals were found between the selected typologies of care when the corresponding absolute medians of the most (fourth quartile) and least (first quartile) efficient services were assessed (Table 5). In T1, the most efficient R1 and R2 services had a greater number of psychologists (*U* = 2.5; *p* = 0.018) and occupational therapists (*U* = 2.5; *p* = 0.014).

**Table 5:** Number of professionals (median) in the most (fourth quartile) and least (first quartile) efficient services

	Hospital acute (T1: R1 and R2)		Residential and non-hospital non-acute (T2: R9, R11, R12 and R13)		Outpatient and non-acute (T3: O8, O9 and O10)	
	1st Quartile	4th Quartile	1st Quartile	4th Quartile	1st Quartile	4th Quartile
Efficiency median	**0.9442**	**0.9866**	**0.8387**	**0.9854**	**0.8280**	**0.9933**
Psychiatrists	2	1	**0**	**0.005**	1.5	4
Psychiatrists in training	0	0	0	0	0	0
GPs	0	0	0	0	0	0
Other doctors	0	0	0	0	0	0
Nurses	12	8	**0.5**	**4**	**3**	**10**
Psychologists	**0.5**	**1**	0	0	0.75	1.7
Social workers	1	1	0	0	0	1.5
Occupational therapists	**0**	**1**	0	0	**0**	**1.8**
Other	8	6	5.5	8	**0**	**3.5**
Overall	24.45	18	**6.5**	**14**	**5**	**25**

The definition of the DESDE codes is provided in Table 1. Elements in bold highlight bilateral statistically significant differences (*p* < 0.05)

In T2, the statistically significant differences were based on psychiatrist and nurse availability. The most efficient services had a greater number of psychiatrists (*U* = 16; *p* = 0.027), nurses (*U* = 9.5; *p* = 0.016) and professionals in total (*U* = 8.5; *p* < 0.013).

In T3, nurses (*U* = 10; *p* = 0.002), occupational therapists (*U* = 20; *p* = 0.005), other healthcare professionals (*U* = 8; *p* = 0.001) and the total number of professionals (*U* = 2.500; *p* < 0.001) were statistically higher in the most efficient group of services that included multidisciplinary teams. The least efficient included just psychiatrists, nurses and/or psychologists.

Globally, the results showed a lack of psychiatrists in training, general practitioners (GPs) and doctors with a different specialization than psychiatry.

Significant differences were also found for rates of professionals per *number of beds* (T1 and T2) or *number of users* (T3) (means and medians, Table 6). In T1, the most efficient hospital services showed greater rates of occupational therapists (*U* = 1; *p* = 0.012) and higher rates of psychologists and social workers. In T2, the most efficient services had the greatest psychiatrist rate (*U* = 16; *p* = 0.027), which was also relevant to the higher nurse rate. In T3, occupational therapist (*U* = 20; *p* = 0.005) and other healthcare professional (*U* = 22.5; *p* = 0.032) rates were significantly high in the most efficient services, but there were also relevant and higher differences in the rates of psychiatrists, nurses and psychologists.

**Table 6:** Rates of professionals per bed (T1 and T2) or per user (T3) [median (mean)] in the most (fourth quantile) and least (first quartile) efficient services

	Hospital acute (T1: R1 and R2) (Rate per bed × 10)		Residential and non-hospital non-acute (T2: R9, R11, R12 and R13) (Rate per bed × 10)		Outpatient and non-acute (T3: O8, O9 and O10) (Rate per user × 1000)	
	1st Quartile	4th Quartile	1st Quartile	4th Quartile	1st Quartile	4th Quartile
Efficiency median	**0.9442 (0.9258)**	**0.9866 (0.9874)**	**0.8387 (0.8222)**	**0.9854 (0.9814)**	**0.828 (0.8133)**	**0.9933 (0.9935)**
Psychiatrists	1.11 (1.6)	0.83 (1.08)	**0 (0)**	**0 (0.16)**	2.71 (2.84)	3.8 (3.76)
Psychiatrists in training	0 (0.07)	0 (0)	0 (0)	0 (0)	0 (0)	0 (0.38)
GPs	0 (0)	0 (0)	0 (0)	0 (0)	0 (0)	0 (0)
Other doctors	0 (0)	0 (0)	0 (0)	0 (0)	0 (0)	0 (0.11)
Nurses	6.67 (9.56)	6.25 (6.42)	0.29 (0.52)	1.04 (1.19)	6.27 (13.04)	8.18 (9.52)
Psychologists	0.33 (0.37)	0.83 (0.77)	0 (0)	0 (0)	0.96 (1.11)	1.77 (1.88)
Social workers	0.67 (0.64)	0.83 (0.77)	0 (0)	0 (0.1)	0 (0.7)	1.26 (0.98)
Occupational therapists	**0 (0.13)**	**0.83 (0.77)**	0 (0)	0 (0.07)	**0 (0)**	**1.03 (1)**
Other	4.44 (4.04)	5 (4.94)	2.86 (3.17)	2.36 (3.69)	**0 (4.43)**	**2.28 (17.93)**

The definition of the DESDE codes is provided in Table 1. Elements in bold highlight bilateral statistically significant differences (*p* < 0.05)

## Discussion

The aim of this research was to examine usage patterns and technical performance (efficiency) in the main types of MH services in the Helsinki-Uusimaa region, Finland. Additionally, it sought to analyse potential relationships between technical performance and the corresponding workforce structure. The study revealed distinct usage patterns in service management, especially in non-acute non-hospital residential care and non-acute outpatient care. It also found high and very high overall technical performance in the assessed services, with the highest average RTE observed in acute hospital residential care. Workforce composition was identified as playing a crucial role in the technical performance of MH services, highlighting the association of higher RTE in acute hospital residential and non-acute outpatient care services integrated by multidisciplinary teams.

To the best of our knowledge, this is the first study to assess the quantitative technical performance of MH services in a Finnish region. It also includes an analysis of potential usage patterns and workforce capacity in three selected typologies of care.

The identified patterns of use could indicate that the available general recommendations and guidelines are consistent throughout the country (or for some regions), despite the fragmentation of governance and the limitations in management at the municipality level [19, 22, 25]. Hospital services probably have a higher level of autonomy to adapt their resource provision and workforce to local user needs. However, in the Helsinki and Uusimaa region, there was no relationship between the existence of patterns of use (T2 and T3) and service technical performance, which was always high on average. More “flexible” services (T1) showed similar performance to more “guided” ones.

In T1 and T3, the most efficient services were provided by multidisciplinary care teams. These results contradict classical assumptions where lower input rates are associated with higher efficiency scores because the balanced care model has been considered the paradigm to interpreting variable values (raw data must be explained to DEA models in terms of appropriateness to avoid undesirable bias in the analysis). This evidence supports the hypothesis that multidisciplinary teams provide better and holistic MH care while maintaining an appropriate input/output balance supported by a diversity of professional skills and treatments [2, 49].

In T2, the workforce, mainly based on psychiatrists, nurses and other professionals, such as healthcare assistants, allows the manager to escalate the resources according to the specific user and population needs (they have more flexibility to adapt care to their corresponding municipal socio-economic environments) [50]. This process is easier when municipalities provide financial support and structural management but is far behind when a person-centred approach is required for reducing barriers and improving the independence of users [51].

The lower structure (low rate of MH professionals) of community residential care and the low level of high turnover of non-hospital residential services in this category may point to a trans-institutionalization effect in care for severe MH patients in Finland [20, 24, 52], where patients move to mostly private non-hospital residential facilities without removing the barriers to full social integration [53]. This process is also associated with a lack of richness and diversity of MH services, which could translate into lower effectiveness [54]. However, the transfer to community residential care does not imply per se a process of re- or trans-institutionalization. There has been a transfer of inpatients to residential community care in Helsinki-Uusimaa. This transfer would require an in-depth longitudinal analysis of the pathways of care and the quality of care provision in the non-hospital sector to fully understand whether this process is related to trans-institutionalization or to balance supported accommodation, as described and analysed in England [36].

Efficient community-based managerial models for MH ecosystems have been previously described in the Basque Country (Spain) [55, 56] and in England [36]. However, due to the differences in the Finnish framework, specific tailor-made interventions and policies are required to obtain a positive outcome [53]. The results showed that strong variations in service technical performance were expected if major changes in service design and managerial strategies were developed. For example, in Bizkaia (Basque Country, Spain) [43], RTE scores show high variability, but MH services also show a relatively high probability of being efficient [40], while in Helsinki-Uusimaa, the RTE scores were highly homogeneous and the probability of being efficient was very low.

This study is limited by the dataset, which was compiled in 2013 and updated in 2018. Data only include service-level and technical variables, such as workforce structure, number of beds, and capacity, among others. Therefore, DEA models do not provide insights about the service accessibility or the quality of care, variables that should be reported not only by the managers, but also by the users and their families. However, the expert knowledge formalized to interpret variable values before the RTE assessment integrated local insights into when they (variable values) can be considered ‘appropriate’ for delivering quality care, all while considering the specific availability of resources and its context. Additional studies could gather information in a manner similar to the previous research by Killaspy et al. [57, 58]. This approach would enable a broader examination of the Finnish MH care system’s performance, similar to those conducted in England (supported accommodation services) [36].

The geographical distribution of the users and the associated accessibility of their MH services have been demonstrated to play a crucial role in the planning and decision-making processes [59]. The Helsinki-Usimaa region is medium-sized and relatively homogeneous from a MH management point of view, but future researches concerning the Finnish MH system should also include a meso-level approach [41, 60] to incorporate a deeper analysis of the influence of geographical allocation and how they are structured in catchment areas [40, 55, 56].

The methodology used was based on RTE as a key indicator of service technical performance, and the Finnish framework showed a very uniform pattern (many services were very similar). In this situation, it was difficult to discriminate high standard units from those that would need large structural changes to improve. Further studies should conduct comparative efficiency and benchmarking analyses with other areas in Finland and in other countries, taking into account the type of funding and management as well as the user profile (age, sex, diagnosis, etc.) to uncover the potential variability hidden behind these factors.

Finally, the findings of this study underscored the need to differentiate new subtypes of community residential services in the DESDE-LTC coding system, for example, in non-hospital residential services for indefinite stay aimed at moving users to other types of supported accommodation (move-on services).

## Conclusions

Residential care is predominant in the MH system in Helsinki-Uusimaa. This fact could be related to a process of trans-institutionalization and is partly driven by specific socio-economic issues, including a lack of family support. In this complex environment, the present study aims to examine the patterns of use for identifying, if they exist, hidden managerial behaviours in the main types of care to assess the technical performance of comparable services (taking into account the patterns of use) by calculating the RTE of the main types of care, and finally, to identify potential relationships between technical performance and the structure and distribution of the MH service workforce.

The analysis of the Helsinki-Usimaa MH system showed the existence of some clear patterns of use, depending on the main type of care, in service management. These patterns have been highlighted in non-acute non-hospital residential care and in non-acute outpatient care. Strong linear relationships can be interpreted as a response of a relatively stable managerial structure (it follows a set of predefined rules) that results in a resource increase when the number of users also increases (the slope can vary depending on the type of care). Furthermore, acute hospital residential care services did not highlight any relationship between the *number of beds* and *length of stay* and the *number of users*.

In all the selected typologies, the technical performance of the Helsinki-Uusimaa MH ecosystem was high or very high (RTE on average). Acute hospital residential care services showed the highest RTE on average, and according to the specific MH environment analysed, an adequate and very uniform balance among resources and outcomes. Non-acute non-hospital residential care as well as non-acute outpatient care services had a lower RTE on average because the their structural variability in providing MH care is higher.

Considering the relevance of the workforce in MH service efficiency, high-performance services were compared with lower-performance services. The implementation of multidisciplinary teams was efficient regarding the balanced care model for acute hospital residential care and non-acute outpatient care services. In non-acute non-hospital residential care, services were mainly based on psychiatrists, nurses and other professionals, such as healthcare assistants, to supply the users’ needs. The most efficient were those with a greater number of psychiatrists and nurses.

The analysis of the characteristics highlighted for the different main types of care in the Helsinki-Uusimaa region could be useful for (i) analysing potential interventions to improve service performance (the promotion of multidisciplinary teams could be an option for some types of services) and (ii) understanding the behaviour and evolution of a real MH system according to the balanced care model paradigm.

### Supplementary Information

**Additional file 1:** Metadata.

**Additional file 2:** Regression Analysis Dataset.

**Additional file 3:** DSS Dataset.

## Data Availability

The dataset supporting the conclusions of this article is included within the article and its additional files.

## Abbreviations

MH: Mental health
REFINEMENT: REsearch on FINancing systems’ Effect on the quality of MENTal health care
REMAST: REFINEMENT Mapping Services Tool
DESDE-LTC: Description and Evaluation of Services and DirectoriEs for Long-Term Care
GIS: Geographical information systems
ESMS-R: European Service Mapping Schedule-Research
KDD: Knowledge discovery from data
AI: Artificial intelligence
EDeS-MH: Efficient decision support-mental health
DSS: Decision support system
DEA: Data envelopment analysis
RTE: Relative technical efficiency
DMU: Decision-making unit
EbCA: Expert-based cooperative analysis
