# Supplementary material for: Impact of the workforce allocation on the technical performance of mental health services: the collective case of Helsinki-Uusimaa (Finland)
Source: Health Res Policy Syst. 2023 Oct 23;21:108. doi: 10.1186/s12961-023-01061-y (PMC10594770; doi:10.1186/s12961-023-01061-y)
Supplement: Supplementary file 1 — Additional file 1: Metadata. [file 12961_2023_1061_MOESM1_ESM.pdf]

Metadata for "Impact of the Workforce Allocation on the Performance of Mental Health Services: the case of Helsinki-Uusimaa (Finland)"

The mental health services included in these two files are classified according to the *Description and Evaluation of Services and DirectoriEs for Long Term Care DESDE-LTC* (1). This international classification provides a clear coding of services described by their principal or most meaningful activity to avoid ambiguity and facilitate modelling studies in health economics and comparative effectiveness for evidence-informed planning. The codes used in this case are explained in the following table:

**Main Type of Care**	**Main Type of Care Description**	**DESDE-LTC Code**	**Type of Facilities**
Residential care	Hospital, acute, 24 h physician cover, different levels of care intensity	R1, R2	General hospitals, psychiatric hospitals and other specialized hospitals
	Non-hospital, non-acute, different levels of non-medical support and length of stay	R9, R11, R12, R13	Residences, houses, and therapeutic communities with various levels of support
Outpatient care	Non-acute, non-mobile, different levels of care intensity	O8, O9, O10	Community mental health teams, outpatient psychiatric clinics and single-handed psychiatrists and psychologists

This zip directory contains two xlsx files. One was used for regression analysis in order to identify relationships among variables associated with the services (called "Finnish_MH_Regressions_Dataset.xlsx"), and another containing the data to conduct Data Envelopment Analysis in order to obtain the Relative Technical Efficiency of each service (called "Finnish_MH_RTE_Dataset.xlsx"). Both files contain the same sheet, as follows:

1. R1-R2: Variables related to Acute Hospital Residential Care services.

2. R9-R13: Variables related to Non-acute Non-hospital Residential Care services.

3. O8_O10: Variables related to Non-acute Non-mobile Outpatient Care services.

Regression Analysis Dataset (Finnish_MH_Regressions_Dataset.xlsx)

A glossary with the label and description for every variable presented in the sheets can be found below:

Label	Description
DESDE_CODE	Service Types included in the analysis of performance according to the taxonomy and classification of services
Beds	Number of service beds
Days	Number of days of stay of the total service users in a natural year
Users	Number of service users in a natural year
Contacts	Number of visits received in a natural year

Relative Technical Efficiency Dataset (Finnish_MH_RTE_Dataset.xlsx)

This file contains a set of variables expressed on rates used as inputs and outputs in the data envelopment analysis models. A glossary with the label and description for every variable presented in the sheets can be found below:

Label	Description
ID	Identification number
DESDE_CODE	Service Types included in the analysis of performance according to the taxonomy and classification of services
Psychiatrists	Number of psychiatrists in the service per number of beds in the service
PsyTraining	Number of psychiatrists in training in the service per number of beds in the service
Nurses	Number of nurses in the service per number of beds in the service
Psychologists	Number of psychologists in the service per number of beds in the service
SocialWorkers	Number of social workers in the service per number of beds in the service
OccupationalTherap	Number of occupational therapists in the service per number of beds in the service
OtherProf	Number of other professionals (e.g. auxiliary nurses) per number of beds in the service
Users	Number of service users in a natural year per number of beds in the service
Contacts	Number of visits received in a natural year per number of beds in the service (in R1-R2)/users (in O8-O10)

LengthStay	Number of days of stay of the total service users in a natural year per number of service users

Reference

1. Salvador-Carulla L, Alvarez-Galvez J, Romero C, Gutiérrez-Colosía MR, Weber G, McDaid D, et al. Evaluation of an integrated system for classification, assessment and comparison of services for long-term care in Europe: The eDESDE-LTC study. BMC Health Serv Res. 2013;13(1).
